# Gastrocnemius medialis tendon properties do not differ between male academy soccer players and control participants but are related to jump performance

**DOI:** 10.1007/s00421-025-05763-9

**Published:** 2025-04-08

**Authors:** David C. Robshaw, Conall F. Murtagh, Barry Drust, Robert M. Erskine

**Affiliations:** 1 School of Sport and Exercise Sciences, Liverpool John Moores University, Liverpool, L3 3AF UK; 2 School of Sport, Exercise and Rehabilitation Sciences, University of Birmingham, Birmingham, UK; 3 Institute of Sport, Exercise and Health, University College London, London, UK

**Keywords:** Plantar flexors, Achilles, Young’s modulus, Tendon stiffness, Football, Countermovement jump (CMJ), Drop jump

## Abstract

The aims of this study were to investigate (i) differences in gastrocnemius medialis (GM) tendon properties between adult male soccer players from an English category one academy (ASP) and male control participants (CON); and (ii) the relationships between GM tendon properties and jump performance. The GM tendon force–elongation relationship was measured in 13 ASP (mean ± SD height 1.81 ± 0.07 m; mass 73.6 ± 5.4 kg; age 18.8 ± 1.2 years) and 11 CON (1.74 ± 0.05 m; 71.2 ± 7.6 kg; 22.3 ± 3.0 years) using a combination of dynamometry, ultrasonography and electromyography. Participants also performed a series of unilateral and bilateral countermovement jumps (CMJ) and bilateral drop jumps on a force platform. GM tendon properties did not differ between groups but maximal tendon elongation correlated inversely with unilateral vertical CMJ peak power (*r* = −0.427, *P* = 0.037). Furthermore, leg stiffness and peak force during a drop jump correlated with GM tendon stiffness (*r* = 0.431–0.462, *P* = 0.035–0.023), maximal tendon force and Achilles tendon CSA (*r* = 0.409–0.737, P ≤ 0.047). These results suggest that GM tendon properties do not differ between ASP and CON but a stiffer GM tendon may facilitate the production of larger forces to increase power output during a unilateral vertical CMJ. Furthermore, higher force and stiffness outputs are achieved during a drop jump by stronger individuals with larger and stiffer GM tendons, which could lead to greater pitch-based performance and may represent a protective mechanism to shield the tendon against injury.

## Introduction

Maximal acceleration and sprinting are considered to be key performance indicators (KPIs) for academy soccer players (Emmonds et al. 2016). Power and leg stiffness (centre of mass displacement relative to ground reaction force) are key physical qualities required for acceleration and sprinting performance, which can be measured via jump assessments such as countermovement jumps (CMJ) and drop jumps (Bontemps et al. 2024; Maloney et al. 2018; Murtagh et al. 2018). Furthermore, the plantar flexors, in particular the gastrocnemius medialis (GM) muscle–tendon unit (MTU), are integral to jumping and sprinting performance, with significant demands placed on it to generate and transmit large forces over short periods of time to accelerate centre of mass (Dorn et al. 2012; Hamner & Delp 2013; Schache et al. 2019; Wade et al. 2018).

Previous studies have investigated how tendon mechanical properties, measured in vivo, can relate to jumping performance (Foure et al. 2011; Murtagh et al. 2018). The relationship between GM tendon properties and jump performance, however, is inconclusive due to contrasting findings (Kubo et al. 2007a, b; Pentidis et al. 2020; Wu et al. 2010). Several authors have demonstrated positive correlations between GM tendon stiffness and jump performance, although this may depend on the type of jump employed (Kubo et al. 2007a, b; Pentidis et al. 2020; Wu et al. 2010). However, increases in GM tendon stiffness, following isometric training interventions, do not necessarily coincide with improvements in jump height during a CMJ or drop jump (Kubo et al. 2007a, b). Rather, jump heights can increase following plyometric training in the absence of changes to tendon stiffness (Kubo et al. 2007a, b). Thus, inconsistent findings between altered mechanical properties of the GM tendon and changes in jumping performance outputs have led to uncertainty in the relationship between the two variables and therefore require further investigation.

Understanding how GM tendon characteristics might influence the outcome of KPIs in soccer players may help with the design of more effective training interventions. Therefore, the aims of this study were to i) investigate the mechanical and material properties of the GM tendon in a group of adult male soccer players from an English category one academy (ASP) and age-matched recreationally active male control participants (CON); and to ii) investigate relationships between these properties and various measures of jump performance in a series of jumping tasks. It was hypothesised that GM tendon stiffness would be greater in ASP *vs.* CON, and would correlate positively with jump performance during CMJs and drop jumps.

## Methods

### Participants

Twenty-four healthy young men, comprising 13 adult academy soccer players (ASP; age, 19 ± 1 years; height, 1.81 ± 0.07 m; mass, 73.6 ± 5.4 kg; mean ± SD), and 11 recreationally active control participants (CON; age, 22 ± 3 years; height, 1.74 ± 0.05 m; mass, 71.2 ± 7.6 kg) volunteered to take part in this study, which was approved by the University Research Ethics Committee (approval number: 17/SPS/030) and complied with the Declaration of Helsinki. The required sample size was estimated before conducting the study with G*Power software (v. 3.1.9.6, Heinrich-Heine-Universität, Düsseldorf). The a priori estimation was performed using a previously reported correlation (*r* = 0.616; P < 0.001) between patellar tendon strain and peak power during a unilateral CMJ in male academy soccer players from the under 18 to under 23 (U18-U23) age groups (Murtagh et al. 2018). A minimum of 20 participants was deemed necessary to detect a significant correlation (*α*: 0.05; *β*: 0.90). However, to account for a potential 20% participant withdrawal from the study, we recruited 24 participants to ensure our study would remain statistically powered. ASP all participated in either U23 or U18 level soccer at an English Premier League Category One soccer academy, while CON were included if they undertook 3–4 h per week of recreational sporting activities, including soccer training. All participants were free from any lower limb injury within the past 3 months and provided written informed consent to participate in this study.

### Experimental design

**Fig. 1:**
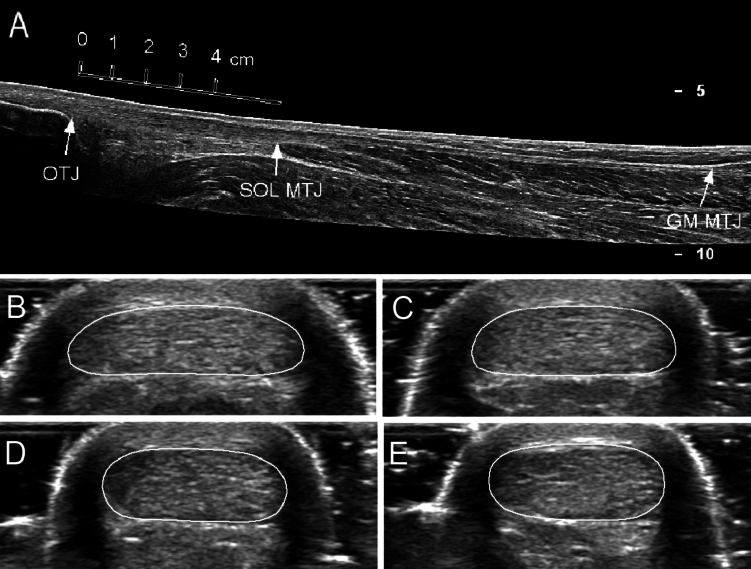
Representative extended field of view (EFOV) ultrasound image of the GM tendon length from distal GM myotendinous junction (GM MTJ) to osteotendinous junction (OTJ), along with the ‘free’ tendon length from the OTJ to the distal soleus MTJ (SOL MTJ), where CSA images were taken (**A**); and ultrasound transverse CSA images of the Achilles tendon at 1 (**B**), 2 (**C**), 3 (**D**) and 4 (**E**) cm proximal to the OTJ, in which the tendon CSA perimeter has been highlighted

Participants attended the laboratory on two separate occasions at least 48 h apart. The initial session familiarised participants with all relevant testing procedures, which included at least 3 plantar flexion (PF) and dorsiflexion (DF) maximal voluntary isometric contractions (MVCs) of the dominant leg (their preferred kicking leg) on an isokinetic dynamometer (IKD). Additional 6 s ramped PF MVCs were performed. After this, participants were familiarised with a series of bilateral and unilateral (dominant limb only) vertical CMJ, horizontal CMJ and (bilateral only) vertical drop jumps. Participants performed at least 3 trials for each jump protocol. During the second session (at least 48 h post-intensive activity), with the participants relaxed in the prone position and their ankle set at 0° (neutral), ultrasound images were taken in the sagittal plane along the length of the gastrocnemius (GM) tendon to measure its length (Fig. 1). Furthermore, transverse cross-sectional area (CSA) images at 1 cm intervals along the Achilles tendon from the distal osteotendinous junction (OTJ) were also captured. Participants were then seated on the IKD and produced 3 PF MVCs, 3 DF MVCs, 3 × 6 s ramped PF MVCs and several passive ankle rotations with an ultrasound probe positioned over the distal GM myotendinous junction (MTJ). Surface electromyography (EMG) electrodes recorded agonist (GM) and antagonist (tibialis anterior, TA) muscle activity during all MVCs. Participants then performed a standardised warm-up, which involved jogging, dynamic movements and stretches, before completing three practice trials of each jump type. Following this, participants completed three trials of each jump protocol on a force plate, recording vertical, medio-lateral, and anterior–posterior ground reaction forces.

### Tendon CSA and length

Participants remained relaxed in the prone position on a therapy bed with the ankle set to the neutral position. A 40 mm wide ultrasound probe (18–5 MHz) was placed on the skin in the sagittal plane, identifying both the distal MTJ of the GM and the OTJ. Water-soluble gel was applied to the surface of the probe and to the skin and an extended field of view (EFOV) sagittal image (Phillips Epiq 7, Guildford, UK) was taken from the distal GM MTJ to the OTJ insertion point along the midline of the tendon (Fig. 1). Minimal but constant pressure was applied to the probe to ensure continuous contact with the skin while avoiding compression of soft tissue. An anthropometric tape measure was used to identify four contiguous 1 cm intervals from the OTJ and a transverse ultrasound image was captured twice at each interval with the probe perpendicular to the skin (Fig. 1). Tendon length and CSA were manually measured using ImageJ (NIH Image, Bethesda, MD, USA). All ultrasound measurements were conducted by the same operator.

### MVC and GM tendon force

Participants were seated and firmly secured on an IKD (Humac Norm, CSMI, Massachusetts, USA), sat in an upright position (hip angle at 85°; supine position = 180°) with their knee fully extended (0° knee joint angle) and ankle set in a neutral (0°) position (90° = full plantar flexion). The centre of rotation on the dynamometer lever arm was in line with the centre of the participants’ lateral malleolus. With the foot fixed in a foot plate attachment, secured by three inextensible straps, the thigh and torso were also secured to the seat with inextensible straps, to minimise additional movement or contribution to torque production from anything other than the plantar/dorsiflexor muscles. Participants completed a standardised warm-up, comprising 10 isokinetic contractions at 60°·s^−1^, gradually increasing contraction intensity. Following the warm-up, participants completed three plantar flexion and three dorsiflexion isometric MVCs with the ankle set at neutral. Each trial alternated between plantar flexion and dorsiflexion, interspersed with 30 s rest and each MVC lasted for ~ 3 s until a plateau in the torque-time trace occurred.

Achilles tendon force (*F*_t_) was calculated by dividing PF MVC torque (corrected for antagonist co-activation, see below for details) by the Achilles tendon moment arm (*d*_AT_, see below for details). Based on the relative proportion of GM muscle volume to total PF muscle volume (Fukunaga et al. 1996), the contribution of the GM muscle to *F*_t_ (i.e. GM *F*_t_) was assumed to be 20.3%. Therefore, GM *F*_t_ was calculated using the following equation:

$${\text{GM}}F_{{\text{t}}} \, = \,\left( {{\text{PF MVC }}/d_{{{\text{AT}}}} } \right) \times 0.{2}0{3}$$

### Tendon elongation (ramped MVC)

Participants were seated on the IKD as per the MVC protocol and previous sub-maximal contractions and MVCs acted as pre-conditioning for the tendon (Maganaris 2003). To assess GM tendon displacement at the MTJ, the probe was placed on the skin, sagitally along the length of the muscle/tendon (over the MTJ, which was stretched sagitally in the proximal direction). To measure the distance the MTJ moved, a strip of 2 mm wide echo-absorbent micropore tape (3 M) was placed on the skin transversely across the length of the tendon, approximately 1 cm proximal to the distal GM MTJ. With the micropore tape ‘shadow’ in the field of view, participants were instructed to produce a graded (ramped) isometric contraction, progressively increasing torque output at a constant loading rate from rest to MVC 6 s later. This movie file was synchronised with the torque-time and EMG-time data using an electrical square wave impulse that was visible simultaneously on both the ultrasound movie file (via the ECG channel) and the AcqKnowledge (Version 5, Biopac Systems Inc., Goleta) moment/angle/EMG data collection file. Ultrasound video clips and torque-EMG traces were checked immediately after each ramped MVC, and if there were any issues (e.g. probe movement), the process was repeated. Once a satisfactory attempt was captured with the ultrasound, it was repositioned over the Achilles tendon distal insertion into the calcaneus. Micropore tape was placed transversely across the Achilles tendon at 2 cm proximal to the calcaneus insertion point, and the above process was repeated to assess distal GM tendon displacement.

### Achilles tendon moment arm

Following tendon elongation and MVCs, thus ensuring the tendon was preconditioned, the Achilles tendon moment arm at 0° was determined using the tendon excursion method (Fath et al. 2010). Participants remained seated as per previous conditions whilst their ankle was passively rotated through + 5° (plantar flexion) to −5° (dorsiflexion) at a speed of 1°∙s^−1^. The ankle was rotated a minimum of three times, at least twice from dorsiflexion to plantar flexion and at least once from plantar flexion to dorsiflexion. Concurrent ultrasound video clips were recorded during these rotations, with the ultrasound probe positioned sagitally over the distal MTJ of the GM where a 2 mm wide strip of echo-absorbent micropore tape was placed transversely on the skin, approximately 1 cm proximal to the distal GM MTJ as a point of reference. Participants were instructed to remain relaxed and allow the rotation to occur without actively producing plantar- or dorsiflexion torque. Ultrasound video clips and torque-EMG-time traces were checked to ensure no movement of the ultrasound probe occurred along with the absence of torque production.

### (Ant)agonist muscle (Co-)activation

Electrical activity of the GM and tibialis anterior (TA) muscles was measured during all MVCs and passive rotations. Following appropriate skin preparation (Hermens et al. 2000), two bipolar (Ag/Ag–Cl) EMG electrodes (Neuroline, Ambu, Ballerup, Denmark) were positioned 20 mm apart along the midline of the muscle, at a third of the muscle length proximally (Hermens et al. 2000). An additional (reference) electrode was positioned on the lateral tibial condyle. EMG activity was sampled using a wireless transmitter (Bionomadix, 2000 Hz, Biopac Systems Inc., Goleta, USA) and recorded simultaneously in AcqKnowledge along with the torque-time trace.

### Jump assessments

Participants performed a series of vertical and horizontal CMJs both bilaterally and unilaterally. Bilateral CMJs were performed in line with current recommendations (Owen et al. 2014), whilst unilateral CMJ protocols have been described in detail elsewhere (Murtagh et al. 2017). The CMJ trial that achieved the highest or furthest distance was used in the following analysis. Participants performed bilateral drop jumps from a 40 cm box, aiming to jump as high and as fast as possible (ground contact time was required to be below 250 ms). Reactive strength index (RSI) was calculated in line with previous guidelines, where jump height (in metres) was divided by ground contact time (in seconds) (Flanagan & Comyns 2008). For the drop jumps, the trial with the highest RSI was used for the following analysis.

### Data processing

#### MVCs

For all plantarflexion and dorsiflexion isometric MVCs, torque was recorded using AcqKnowledge software. For the analysis of peak torque, resting baseline torque values were subtracted from the peak torque recorded across trials with the highest peak torque taken as the MVC torque for both plantarflexion and dorsiflexion. During the ramped isometric MVCs, peak torque was calculated using the same method, where 10% increments in torque were determined and added onto the resting baseline to allow for accurate identification of time occurrence. Ankle moment and angle data were recorded via an analogue-to-digital converter (MP160, Biopac Systems Inc., Goleta, USA), sampled at 2000 Hz with a PC using AcqKnowledge. Torque and angle data were filtered using a low-pass filter at a 10-Hz edge frequency. The EMG signal was filtered using a band-pass filter set at 10 and 500 Hz. The root mean square (RMS) of the EMG signal (calculated every 200 ms) was averaged over 500 ms around (250 ms on either side) peak torque for GM and TA during both PF and DF MVCs. During the ramped MVCs, the TA RMS EMG was averaged over 50 ms at every 10% PF MVC interval. Antagonist (TA) torque contribution during PF was determined and PF MVC was corrected to account for antagonist co-activation using the following equation:

$$({\text{TA}}\;{\text{EMG}}\;{\text{RMS}}\;{\text{PF}}/{\text{Peak}}\;{\text{TA}}\;{\text{EMG}}\;{\text{RMS}}\;{\text{DF}}) \, \times {\text{DF}}\;{\text{Peak}}\;{\text{Torque}})\, + \,{\text{PF}}\;{\text{Peak}}\;{\text{Torque}}$$

#### Calculation of tendon properties

Individual ultrasound movie frames were extracted at each 10% torque increment for the respective trial, with the distance between MTJ and reference mark measured, along with the distance from OTJ to reference marker. MTJ and OTJ displacement were calculated by subtracting the distance between the respective tendinous junction and its relevant reference marker at rest, from the distance between them at each time interval. Following this, torque was corrected by accounting for TA co-activation (see equation used during MVC) and then converted to force by dividing torque by the average moment arm between the two testing occasions. Force–elongation data were then plotted where individual force–elongation curves for MTJ and OTJ displacement were fitted with a second-order polynomial (*R*^2^ > 0.95 in all cases).

To account for internal joint rotation and differences in torque output caused by measurements occurring across separate trials for MTJ and OTJ displacement, the polynomial function for distal displacement was subtracted from the proximal polynomial to calculate overall tendon elongation (Maganaris 2005). This overall tendon displacement was fitted to a new force–elongation curve with a second-order polynomial. Mechanical stress was determined by dividing tendon force by mean tendon CSA. Tendon strain was defined as the elongation recorded during the ramped MVC (proximal minus distal displacement), divided by resting GM tendon length. Young’s modulus was calculated by dividing stress by strain. Stiffness was calculated as the change in force divided by the change in tendon elongation at the absolute forces equating to 80–100% of the weakest participant’s ramped MVC.

#### Achilles tendon moment arm

Ankle joint torque and angle data were recorded in AcqKnowledge and filtered with a 10 Hz low-pass filter. Ultrasound movie files were synchronised with the ankle displacement–time and torque data, as described above, and MTJ displacement (distance between MTJ and tape) was analysed using ImageJ software, as detailed above. A linear trendline was applied to the MTJ displacement and ankle joint rotation data, which was then differentiated for 0° to provide the Achilles tendon moment arm at the same ankle joint angle as the MVCs. Due to the assumption with the tendon excursion method, where no internal force (through active muscular contraction or contribution through friction) is generated during passive joint rotation, equilibrium equations can be determined through the principle of virtual work (An et al. 1984; Storace & Wolf 1979).

### Jump data analysis

Ground reaction force (GRF) data were recorded during all jumps using AcqKnowledge. A customised Excel Spreadsheet (Office 2007, Microsoft, Redmond, USA) was used to calculate jump height, peak power and take-off velocity for vertical CMJ; projectile range for horizontal CMJ; contact time, flight time, RSI and stiffness (centre of mass displacement relative to GRF) during drop jumps. All parameters were calculated using equations described elsewhere (Blazevich 2007; Dowling & Vamos 1993; Flanagan & Comyns 2008; Grimshaw et al. 2004; Moir 2008; Owen et al. 2014). However, peak power was calculated as the sum of instantaneous power recorded for each vector (vertical, anterior–posterior and medio-lateral) (Vigotsky et al. 2019):

$$P\, = \,F_{x} \times V_{x} \, + \,F_{y} \times V_{y} \, + \,F_{z} \times V_{z}$$

### Statistical analysis

All variables were assessed to determine whether they met the parametric assumptions using a Levenes test for homogeneity of variance (P < 0.05) and a Shapiro–Wilk test to determine normal distribution (P < 0.05). If the data were normally distributed, differences between group means were determined using independent samples t tests. If data were not normally distributed, a Mann–Whitney-U independent test was used to compare between-group differences. To compare between (ASP vs CON) and within (location) group differences in Achilles tendon CSA, a mixed ANOVA was implemented. Pearson’s correlation coefficient was used to identify the relationships between tendon properties and jump performance.

A sub-sample of 10 participants attended the laboratory for a third time (2–7 days following the second visit) for the purpose of determining between-session reproducibility for all variables. Reproducibility was assessed using a two-way random effects model (absolute agreement) intraclass correlation coefficient (ICC, including 95% confidence intervals, CI), as well as ratio limits of agreement (RLoA) (Nevill & Atkinson 1997). Absolute reliability was reported as a typical error (TE), while relative reliability was calculated using the coefficient of variation (CV, %) (Hopkins 2000). TE was calculated using the following equation:

Standard deviation of differences/(√2)

CV was determined using the following equation:

$${1}00 \times \, \surd \left( {{{\left( {{{\text{Sum of squared differences}} \mathord{\left/ {\vphantom {{\text{Sum of squared differences}} {\left( {2 \times {\text{ sample size}}} \right)}}} \right. \kern-0pt} {\left( {2 \times {\text{ sample size}}} \right)}}} \right)} \mathord{\left/ {\vphantom {{\left( {{{\text{Sum of squared differences}} \mathord{\left/ {\vphantom {{\text{Sum of squared differences}} {\left( {2 \times {\text{ sample size}}} \right)}}} \right. \kern-0pt} {\left( {2 \times {\text{ sample size}}} \right)}}} \right)} {\left( {\text{Average of measurement 1 and 2}} \right)}}} \right. \kern-0pt} {\left( {\text{Average of measurement 1 and 2}} \right)}}} \right)$$

Unless otherwise stated, data are reported as mean and standard deviation (SD) for each independent variable on each testing occasion. All statistical analyses were completed using SPSS software (Version 26, IBM SPSS Inc., Chicago, USA).

## Results

### Reliability

Low CVs and TEs were found for the majority of Achilles tendon morphological parameters, whilst CSA at 1 cm displayed a higher CV and TE (Table 1). Inter-day ICC values for tendon properties were also strong with narrow 95% confidence intervals for the majority of measures. All tendon parameters, apart from the aforementioned, showed little bias (1.00–1.01) and satisfactory RLoA (*/ ÷ 1.04–1.08). Therefore, there is a 95% chance that any re-assessment of these characteristics would be within ± 8% of the original value. Free AT CSA measured at 1 cm displayed poor agreement ratios (1.27). Achilles tendon moment arm demonstrated a moderate ICC (0.59) with wide 95% confidence intervals (−0.250–0.877, *P* = 0.064), along with a poor agreement ratio (*/ ÷ 1.39), despite a small bias (1.08), thus suggesting low reproducibility. Between-session reliability for GM tendon mechanical properties are displayed in Table 2 and descriptions of the data are provided below.

**Table 1:** Test–retest reproducibility of Achilles tendon morphological characteristics

Tendon characteristic	ICC	95% CI		*P* Value	MBR (*/ ÷ RLoA)	CV (%)	TE (units)
		Lower	Upper				
1 cm CSA	0.845	0.443	0.956	0.003	1.00 (1.27)	8.45	0.06 (cm^2^)
2 cm CSA	0.856	0.491	0.959	0.002	1.01 (1.15)	4.82	0.03 (cm^2^)
3 cm CSA	0.976	0.921	0.993	< 0.001	1.01 (1.07)	2.41	0.01 (cm^2^)
4 cm CSA	0.989	0.961	0.997	< 0.001	1.00 (1.06)	2.02	0.01 (cm^2^)
Mean CSA	0.974	0.91	0.992	< 0.001	1.01 (1.08)	2.61	0.02 (cm^2^)
GM TL	0.993	0.975	0.998	< 0.001	1.00 (1.04)	1.21	0.45 (cm)
*d*_AT_	0.59	−0.25	0.877	0.064	1.08 (1.39)	12.91	0.25 (cm)

*CI* confidence interval; *MBR* mean bias ratio; *RLoA* ratio limits of agreement; *CSA* cross-sectional area; *AT* Achilles tendon; *ICC* intraclass correlation coefficient; *CV* coefficient of variation; *TE* typical error; *TL* tendon length; *d*_AT_ Achilles tendon moment arm

**Table 2:** Test–retest reproducibility of gastrocnemius medialis (GM) tendon mechanical and material properties

Variable	ICC	95% CI		*P* Value	MBR (*/ ÷ RLoA)	CV (%)	TE (Units)
		Lower	Upper				
Stiffness	0.959	0.790	0.993	0.001	1.04 (1.77)	13.5	117 N∙mm^−1^
Stress	0.993	0.960	0.999	< 0.001	1.00 (1.08)	2.58	0.56 MPa
Strain	0.916	0.425	0.986	0.001	1.13 (1.37)	12.3	1.08 %
Elongation	0.897	0.269	0.983	0.002	1.13 (1.33)	12.3	1.95 mm
Young's modulus	0.948	0.721	0.991	0.001	1.04 (1.80)	13.8	181 MPa

*ICC* intraclass correlation coefficient; *CV* coefficient of variation; *TE* typical error; *CI* confidence interval; *MBR* mean bias ratio; *RLoA* ratio limits of agreement

Between-session reproducibility of all jump variables is presented in Table 3. CV analysis for all CMJ variables revealed good to low values for all parameters (2.77–11.38%). Weak-to-strong ICCs with varying 95% confidence intervals were found for all CMJ variables, where peak power achieved statistical significance for all CMJ (*P* ≤ 0.031) except during unilateral horizontal jumps. Small mean bias was present for all jumps (0.97–1.05), whilst RLoA for CMJs range from ± 9–40%. Inter-session reproducibility values for jump height, flight time and contact time during vertical drop jumps produced the lowest CVs amongst all variables (5.35–9.91%), whilst RSI, vertical stiffness and peak force demonstrated higher CVs (10.2–21.5%). However, only vertical stiffness and peak force displayed strong ICC with narrow confidence intervals and significant a *P* value (*P* < 0.001). In contrast, leg stiffness exhibited the greater RLoA (± 65%) of all the parameters.

**Table 3:** Test–retest reproducibility of jump parameters

Jump	Variable	ICC	95% CI		*P* Value	MBR (*/ ÷ RLoA)	CV (%)	TE (units)
			Lower	Upper				
Bilateral VCMJ	Take-off velocity	0.391	−1.358	0.830	0.224	0.99 (1.14)	4.44	0.13 m/s
	Peak power	0.707	0.02	0.915	0.027	1.05 (1.40)	11.38	3.43 W/kg
	Jump height	0.402	−1.281	0.832	0.215	0.98 (1.29)	8.86	3.70 cm
Unilateral VCMJ	Take-off Velocity	0.941	0.804	0.983	< 0.001	1.02 (1.13)	4.33	0.09 m/s
	Peak power	0.980	0.931	0.994	< 0.001	0.97 (1.29)	7.26	1.19 W/kg
	Jump height	0.96	0.868	0.988	< 0.001	1.05 (1.29)	8.16	1.87 cm
	Resultant take-off velocity	0.857	0.51	0.959	0.002	1.01 (1.09)	3.22	0.12 m/s
Bilateral HCMJ	Peak power	0.633	−0.385	0.897	0.031	1.01 (1.26)	8.48	3.16 W/kg
	Projectile range	0.843	0.460	0.955	0.003	1.02 (1.20)	6.99	9.34 cm
	Measured distance	0.932	0.77	0.980	< 0.001	0.99 (1.09)	2.77	5.27 cm
	Resultant take-off velocity	0.606	−0.489	0.889	0.080	0.99 (1.16)	5.22	0.16 m/s
Unilateral HCMJ	Peak power	0.676	−0.209	0. 908	0.337	0.99 (1.36)	11.11	2.55 W/kg
	Projectile range	0.574	−0.631	0.880	0.099	0.99 (1.37)	11.0	10.2 cm
	Measured distance	0.952	0.839	0.986	< 0.001	0.99 (1.17)	4.69	6.54 cm
Bilateral Drop Jump	Jump height	0.632	−0.320	0.895	0.062	1.01 (1.40)	9.91	3.38 cm
	Contact time	0.658	−0.287	0.904	0.053	1.00 (1.25)	7.98	0.02 s
	Flight time	0.566	−0.658	0.878	0.103	1.01 (1.18)	5.35	0.03 s
	Reactive strength index	0.628	−0.361	0.894	0.066	1.01 (1.39)	10.2	0.27 AU
	Peak force	0.926	0.740	0.979	< 0.001	1.03 (1.47)	21.5	539 N
	Vertical stiffness	0.945	0.814	0.984	< 0.001	1.01 (1.65)	18.7	85.71 (N·m·kg^−1^)

*CI* confidence interval; *MBR* mean bias ratio; *RLoA* ratio limits of agreement; *ICC* intraclass correlation coefficient; *CV* coefficient of variation; *TE* typical error

### Gastrocnemius medialis tendon morphological and mechanical properties

Mechanical and material properties of the GM tendon for ASP and CON participants are displayed in Table 4. Results from the mixed ANOVA for tendon CSA are presented in Table 4; no significant differences were observed between- or within groups at any location. In fact, no differences were observed between the two groups for any tendon variable (*P* > 0.05).

**Table 4:** Comparison of GM tendon morphological, mechanical and material properties in academy soccer players (ASP) and recreationally active control participants (CON). Values are mean (SD)

Variable (units)	ASP (*n* = 13)	CON (*n* = 11)	*P* Value
1 cm CSA (mm^2^)	76.7 (13.1)	73.6 (11.0)	0.533
2 cm CSA (mm^2^)	73.5 (13.1)	68.0 (7.6)	0.235
3 cm CSA (mm^2^)	66.3 (10.2)	61.8 (7.3)	0.242
4 cm CSA (mm^2^)	61.6 (10.6)	58.9 (8.3)	0.488
Mean CSA (mm^2^)	69.6 (10.8)	65.6 (7.8)	0.319
GM tendon length (cm)	20.8 (2.4)	19.4 (2.0)	0.090
Tendon moment arm (mm)	30.9 (5.4)	34.2 (4.8)	0.773
Maximum tendon force (N)	1334 (235)	1260 (307)	0.165
Stiffness (N/mm)	824 (223)	789 (260)	0.664
Elongation (cm)	1.14 (0.29)	1.31 (0.43)	0.087
Strain (%)	5.47 (1.36)	6.80 (2.40)	0.060
Young's modulus (GPa)	1.28 (0.52)	1.12 (0.26)	0.885
Stress (MPa)	11.2 (1.7)	11.7 (1.43)	0.461

*CSA* cross-sectional area. Tendon stiffness, elongation, strain, stress and Young's modulus were all measured at the highest common force

**Fig. 2:**
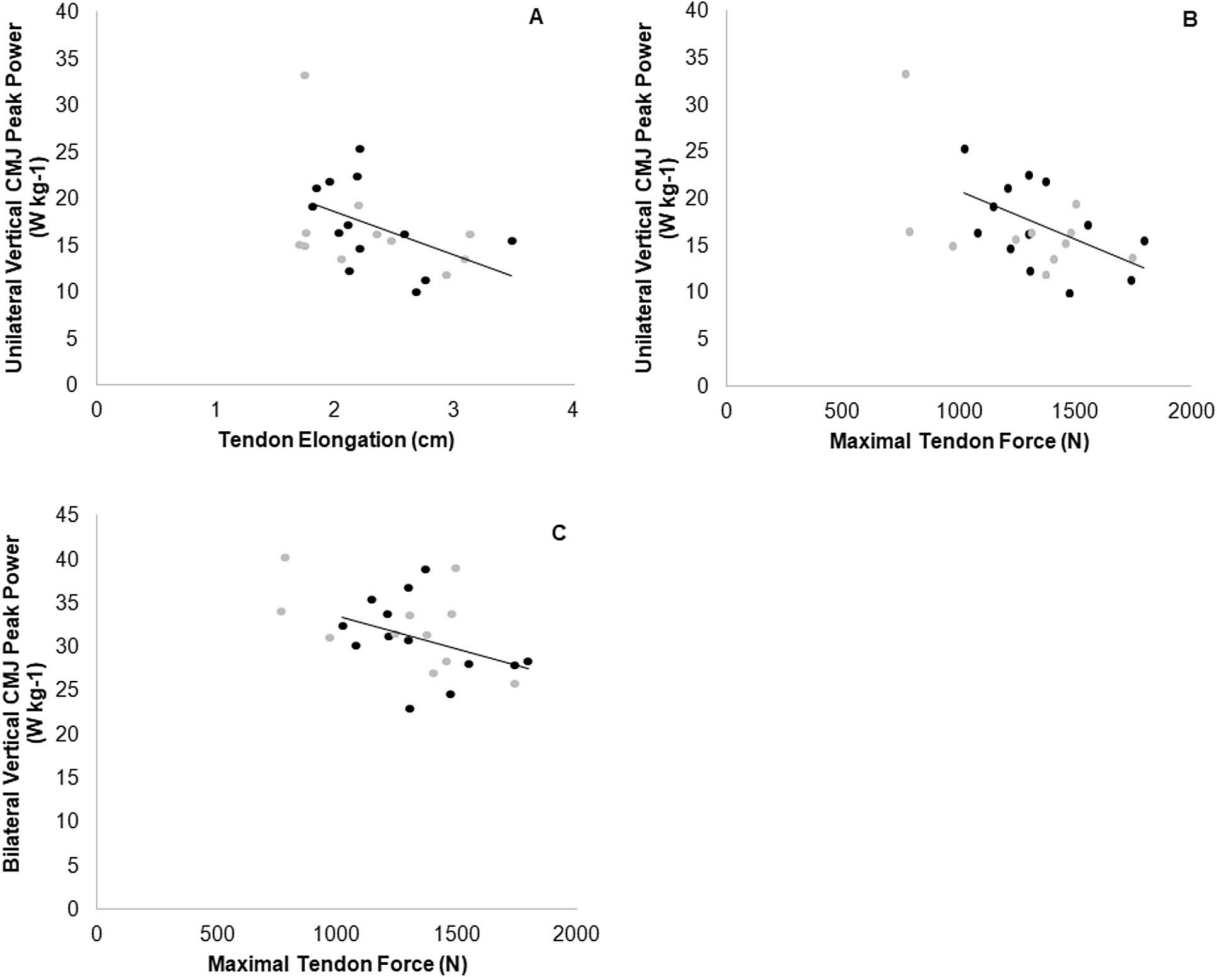
Relationship between unilateral vertical CMJ peak power with maximal tendon elongation (**a**, *r* = −0.427, *P* = 0.037) and maximal individual force (**b**, *r* = −0.435, *P* = 0.033), and bilateral vertical CMJ peak power with maximal individual force (**c**, *r* = −0.461, *P* = 0.023), in ASP (black data points) and CON (grey data points)

**Fig. 3:**
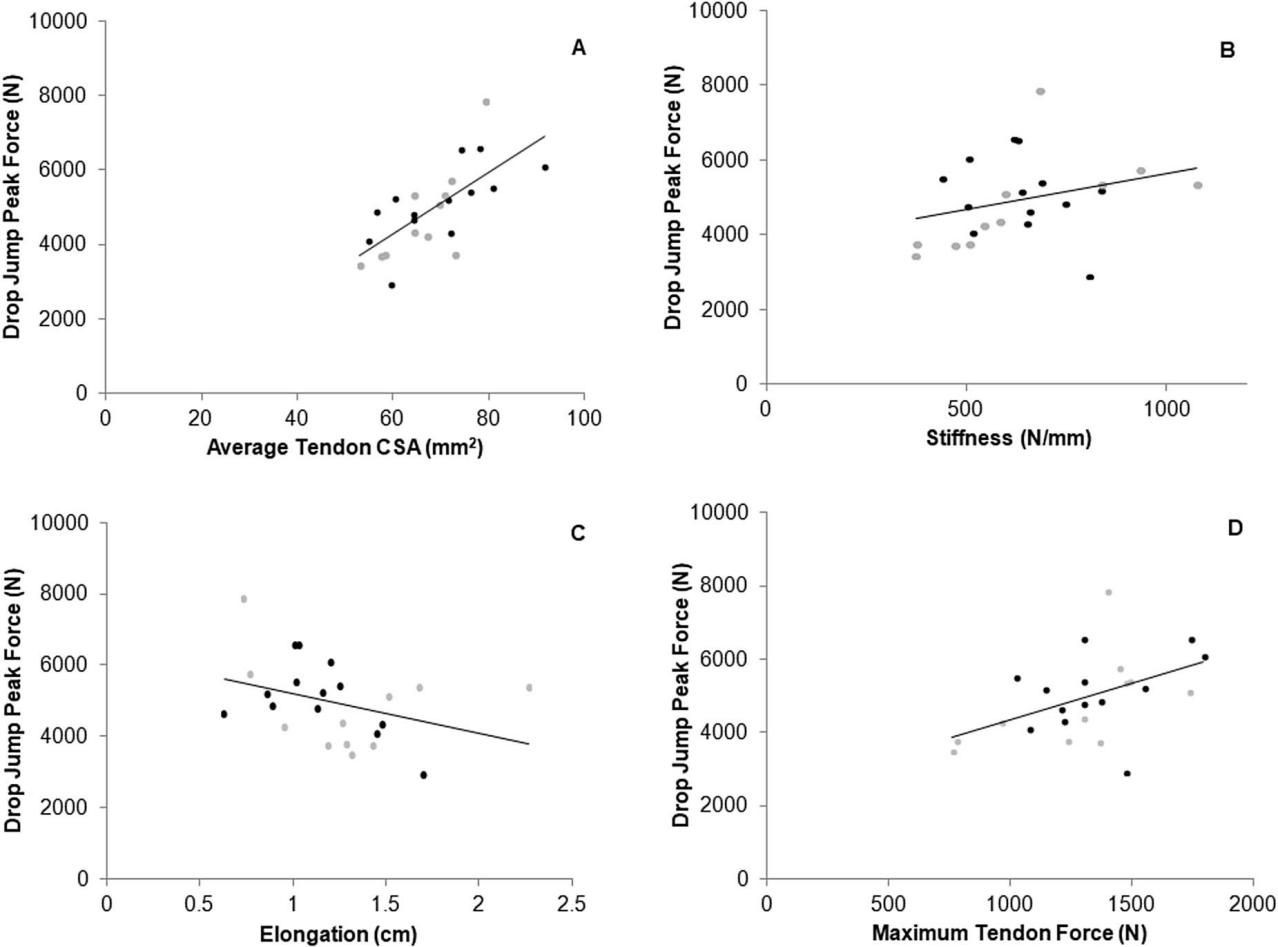
Relationship between vertical drop jump peak force and average tendon cross-sectional area (CSA) (**A**, *r* = 0.737, *P* < 0.001), tendon stiffness (**B**, *r* = 0.431, *P* = 0.035), elongation over a common force range (**C**, *r* = -0.416, *P* = 0.043) and maximal individual force (**D**, *r* = 0.501, *P* = 0.013) in ASP (black data points) and CON (grey data points)

**Fig. 4:**
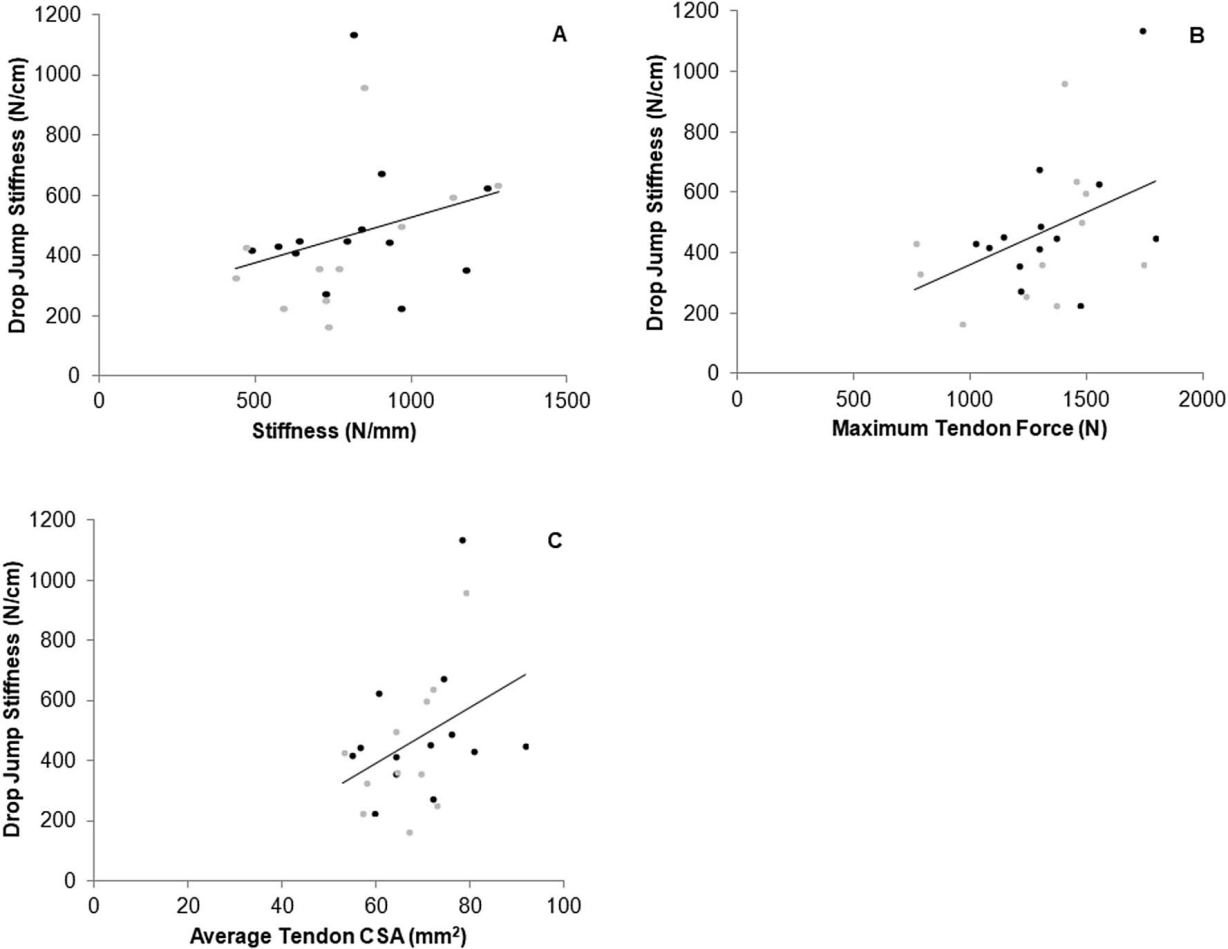
Relationship between vertical drop jump stiffness and stiffness over a common force range (**A**, *r* = 0.462, *P* = 0.023), maximal individual force (**B**, *r* = 0.441, *P* = 0.031) and average tendon cross-sectional area (CSA) (**C**, *r* = 0.409, *P* = 0.047) in ASP (black data points) and CON (grey data points)

Correlations (including Pearson’s r and corresponding P values) between GM tendon properties and jump performance variables are presented in Figs. 2, 3, 4. Inverse correlations were found between peak vertical power and peak tendon force during bilateral and unilateral vertical CMJ. Similarly, unilateral vertical CMJ peak power correlated inversely with tendon elongation, while stiffness during a drop jump correlated positively with tendon stiffness, maximum tendon force and stress. No significant relationships were found between any horizontal CMJ and tendon properties (*P* > 0.05).

## Discussion

The aims of this study were to investigate (i) whether the mechanical and material properties of the GM tendon differed between a group of ASP and CON; and (ii) if GM tendon properties were related to power and force variables during different CMJs and drop jumps. We found that GM mechanical and material properties did not differ between ASP and CON. However, several key correlations between tendon properties and jump performance were found, whereby maximal elongation of the GM tendon correlated inversely with unilateral vertical CMJ peak power, while tendon stiffness, maximum force and tendon CSA all correlated positively with peak force and stiffness during a bilateral drop jump. This study has therefore identified that ASP do not possess distinct GM tendon mechanical and material properties from those of young, healthy, recreationally active males. However, a larger, stiffer GM tendon may be required to provide a protective mechanism during high GRF activities and enable greater lower-limb stiffness capabilities.

In accordance with previous investigations into the strain properties of the GM in different athletic populations (Arampatzis et al. 2007; Wiesinger et al. 2016), the current study found no difference between ASP and CON. This may be expected, as no change in GM tendon strain or elongation has been reported following a range of training interventions (Foure et al. 2011; Geremia et al. 2018; Kubo et al. 2017). In addition, we found no differences in tendon stiffness between ASP and CON. Despite a wide variety of approaches utilised to determine GM tendon stiffness in previous studies (making it difficult to compare between studies), differences in GM tendon stiffness have been reported across athletic populations by Arampatzis et al., (2007). Higher stiffness values coincided with larger plantar flexion forces, whereby 67% of the variance in tendon stiffness reported was associated with maximal tendon force. This suggests that a change in stiffness occurs in response to a change in the force-generating capacity of the muscle. In fact, Seynnes et al. (2009) and Bontemps et al. (2024) found that the change in patellar tendon stiffness after nine weeks’ resistance training (Seynnes et al. 2009) or four weeks’ downhill running training (Bontemps et al. 2024) correlated with the change in muscle PCSA (which represents the maximum force generating capacity of the muscle). In our study, the similar plantar flexor strength and maximal tendon force between the two groups could explain why no difference in tendon properties were found between ASP and CON. The similarity in plantar flexion peak torque between groups may also demonstrate the limited ability of pitch-based soccer training to elicit improvements in GM neuromuscular strength, although this remains to be investigated.

Previous studies have investigated the relationship between tendon properties and jump height, with contrasting findings, but few studies have examined correlations with power or stiffness outputs (Kubo et al. 2007a, b; Pentidis et al. 2020; Wu et al. 2010), which should be the focus of jump assessments (Murtagh et al. 2017). In the current study, an inverse correlation was found between peak power during a unilateral vertical CMJ and GM tendon elongation. This suggests that a stiffer GM tendon provides a more efficient transference of force, thus leading to greater power outputs.

As power is the product of force × velocity, a less extensible tendon would likely coincide with either a greater ability to transfer force or an improved ability to recoil at faster velocities. Considering our findings, it would be logical to assume that, for a given strain to be achieved in a stiffer tendon, a larger production of force would be needed than that required for a more compliant tendon. On the other hand, a stiffer tendon may undergo less elongation during the stretch, which would cause the attaching muscle to contract eccentrically (Hicks et al. 2013), generating a larger force. In both scenarios, the energy stored and released by the stiffer tendon would therefore be greater, which would return a more forceful MTU contraction, increasing power output. Similarly, an increase in GM architectural gear ratio, through shortening and rotation of fascicles, could maintain strain in a stiffer tendon (Werkhausen et al. 2019). An increase in this ‘gearing’ of the muscle fascicle will lead to an increased stretch placed on the tendon, thus enabling more energy to be stored and returned. Regardless of the specific mechanisms that influence tendon strain during running and jumping, a stiffer tendon would still be expected to augment power output.

However, the inverse relationships between power output and tendon elongation conflict with previous reports of the patellar tendon, where a more compliant tendon has been associated with power output in similar jump assessments (Murtagh et al. 2018). These findings might represent the innate differences between these two MTUs, as the quadriceps MTU possesses a greater quantity of contractile tissue than the plantar flexors (Wickiewicz et al. 1983), coupled with a shorter, thicker tendon. Whilst power and positive work generated by the GM MTU during running and jumping requires a larger contribution from the tendon than muscle fibres (Bobbert et al. 1986; Lai et al. 2014), the quadriceps might require a greater contribution from the contractile tissue. A more compliant patellar tendon could therefore facilitate the generation of greater forces during a CMJ. It might be surprising that tendon stiffness has not also shown the same relationship as strain, considering stiffness is the result of changes in length relative to changes in force during the linear region of the force–elongation curve. Our findings suggest the ability of the tendon to elongate over these maximal force ranges (80–100% MVC) does not influence power output during a vertical CMJ. Previous research has assessed tendon length change over 50–100% MVC (Fletcher & MacIntosh 2015; Kubo et al. 2017), which may capture significant length changes to the tendon missed by using a higher force range. Should variation be present between populations, a narrow force range might not be subtle enough to detect such differences.

Stiffness over a common force range, along with tendon force, displayed positive relationships with vertical GRF and leg stiffness during a bilateral drop jump. One possible explanation for these findings could be that a stiffer tendon enables muscle fibres to contract isometrically during the jumping action, as previously suggested by Hirayama et al., (2017). Less displacement of the tendon for a given force would reduce the fascicular shortening required, thus enabling larger forces to be generated from a more optimal length-tension position. Alternatively, an eccentric contraction of the GM may also allow less displacement of the tendon, whilst generating higher forces to be returned via elastic energy. Additionally, positive correlations between jump performance and tendon CSA suggest that a larger tendon would be able to withstand higher forces produced by the GM, increasing its safety factor, which serves to augment vertical GRF and in turn, stiffness. In support of this suggestion, Monte and Zamparo, (2019) showed positive correlations between Achilles tendon CSA and stiffness and running speeds during sprinting. Greater CSA may, therefore, provide a greater return of elastic energy, suited more to transferring force and in turn, aiding performance.

Regarding the reliability assessment of all variables in this study, the main findings were that the morphology of the GM tendon can be measured with good to excellent reproducibility. Mechanical properties of the GM tendon can be calculated with varying degrees of reliability, whilst jump performance outputs can be considered more reliable when using unilateral VCMJ and bilateral modalities for HCMJ. Caution should be used when interpreting changes in performance outputs from drop jumps due to the slightly poorer reliability compared to CMJs. When compared with previous research, projectile range displayed similar CVs and ICCs for bilateral HCMJ to those reported by (Meylan et al. 2012) (CVs = 3.8–6.0, ICCs = 0.87–0.96), although RLoA and CV were poor for unilateral HCMJ, the reliability of which has not been investigated previously. Reproducibility of vertical stiffness displayed a very similar CV and ICC to previous reports using comparable methods (Maloney et al. 2018).

We do acknowledge some limitations with this study, first, the ASP cohort comprised U23 and U18 male soccer players from a single English Premier League Category One soccer academy, and may not be representative of U18-U23 male academy soccer players in other academies. We would suggest this research is replicated in other cohorts, including female soccer players, to widen the sample. Second, the control cohort of age-matched males took part in a variety of sports, not just soccer. Whilst this does not enable a direct comparison between ‘elite’ and ‘non-elite’ soccer players, it does allow our ASP to be compared with a more representative sample of physically active, healthy young males.

In conclusion, GM tendon mechanical and material properties do not differ between male academy soccer players and recreationally active male control participants. However, correlations between these properties and performance variables during jumping tasks suggest a less extensible tendon may facilitate greater power outputs during a unilateral vertical CMJ. Similarly, a stiffer and larger GM tendon correlated with higher forces generated during a bilateral drop jump and stiffness parameters, potentially serving as a protective mechanism.

## Practical applications

Strength and conditioning coaches can utilise this information to develop training interventions that target increasing CSA and stiffness of the gastrocnemius medialis tendon to improve lower limb power and leg stiffness during jumping tasks. Furthermore, this research provides a normative data set for academy soccer players to compare with other sporting populations as well as identifying the reliability of assessment methods.

## Data Availability

All data discussed in this article are presented in the tables/figures.
